# Targeting novel anti‐complement drugs to the brain reduces complement activation and synapse loss, and improves cognition in a mouse model of dementia

**DOI:** 10.1002/alz.090708

**Published:** 2025-01-09

**Authors:** Wioleta M Zelek, Ryan J Bevan, Jacqui M Nimo, Maarten Dewilde, Bart De Strooper, Bryan Paul Morgan

**Affiliations:** ^1^ Cardiff University, Cardiff United Kingdom; ^2^ Dementia Research Institute, Cardiff University, Cardiff United Kingdom; ^3^ KU Leuven, Leuven Belgium; ^4^ Dementia Research Institute at University College London, London United Kingdom; ^5^ UK Dementia Research Institute at Cardiff University, Cardiff, South Glamorgan United Kingdom

## Abstract

**Background:**

In the brain as in other organs, complement contributes to immune defence and housekeeping to maintain homeostasis. Sources of complement may include local production by brain cells and influx from the periphery, the latter severely restricted by the blood brain barrier (BBB) in healthy brain. Dysregulation of complement leads to excessive inflammation, direct damage to self‐cells and propagation of injury. This is likely of particular relevance in the brain where inflammation is poorly tolerated and brain cells are vulnerable to direct damage by complement.

**Method:**

We have developed novel anti‐C7 antibodies (mAb) that efficiently inhibit formation of the pro‐inflammatory membrane attack complex (MAC) *in vitro* and *in vivo*. Here we describe recombinant fusion proteins (FP) that replicate the MAC‐blocking action of the mAb, and are designed to access the brain utilising “Trojan horse” shuttles. The Alzheimer model APP^NL‐G‐F^ mice were treated systemically with native mAb to swamp peripheral C7 followed by the FP. Immunohistochemistry and ELISA were used to demonstrate FP entry into brain and show impact on the disease pathology.

**Result:**

The recombinant FP showed complement inhibitory activity *in vitro* equivalent to their parent mAb and were able to cross an artificial BBB in transwells. The presence of the FP in brain homogenates of peripherally dosed animals was confirmed by ELISA. Treatment with the FP caused reduced levels of complement activation products C3b and terminal complement complex (TCC) in brain. Diolistics analysis showed significant increased neuronal spine density in treated mice compared to controls, demonstrating a protective effect of the FP on synaptic function. Mice treated with the drug showed significant improvements in cognition.

**Conclusion:**

The FP described are able to cross BBB and are potent inhibitors of complement in brain; impact on brain pathology was detected after just one week of treatment. The findings highlight the potential for complement inhibition as a therapy in Alzheimer’s disease.

